# Alice in Wonderland Syndrome (AIWS): prevalence and characteristics in adults with migraine

**DOI:** 10.1007/s00415-024-12471-5

**Published:** 2024-05-31

**Authors:** Mira P. Fitzek, Jasper Mecklenburg, Lucas H. Overeem, Kristin S. Lange, Anke Siebert, Paul Triller, Lars Neeb, Jens P. Dreier, Daniel Kondziella, Uwe Reuter, Bianca Raffaelli

**Affiliations:** 1 Department of Neurology, Charité–Universitätsmedizin Berlin, Corporate Member of Freie Universität Berlin, Humboldt-Universität Zu Berlin, and Berlin Institute of Health, Charitéplatz 1, 10117 Berlin, Germany; 2 Clinician Scientist Program, Berlin Institute of Health at Charité (BIH), Berlin, Germany; 3 Helios Global Health, Berlin, Germany; 4 Center for Stroke Research, Charité–Universitätsmedizin Berlin, Corporate Member of Freie Universität Berlin, Humboldt-Universität Zu Berlin, and Berlin Institute of Health, Berlin, GermanyUniversitätsmedizin Berlin, Berlin, Germany; 5 Department of Experimental Neurology, Charité–Universitätsmedizin Berlin, Corporate Member of Freie Universität Berlin, Humboldt-Universität Zu Berlin, and Berlin Institute of Health, Berlin, Germany; 6 Bernstein Center for Computational Neuroscience Berlin, Berlin, Germany; 7 Einstein Center for Neurosciences Berlin, Berlin, Germany; 8 Department of Neurology, Rigshospitalet, Copenhagen University Hospital, Copenhagen, Denmark; 9 Department of Clinical Medicine, University of Copenhagen, Copenhagen, Denmark; 10 Universitätsmedizin Greifswald, Greifswald, Germany; 11 Junior Clinician Scientist Program, Berlin Institute of Health at Charité (BIH), Berlin, Germany

**Keywords:** Cortical spreading depolarization, Migraine aura, Headache, Visual phenomena

## Abstract

**Objective:**

Alice in Wonderland Syndrome (AIWS) is a sensory disorder characterized by a distorted somatosensory and/or visual perception. Additionally, distortion of time perception and symptoms of derealization/depersonalization may occur. AIWS is frequently associated with migraine. However, its prevalence, and clinical characteristics remain poorly understood. Here, we investigated the prevalence and features of AIWS in individuals with migraine. We hypothesized AIWS is more frequent in migraine patients with aura than in those without aura.

**Methods:**

This was a prospective cross-sectional cohort study, conducted at a tertiary headache center. Participants with migraine filled out questionnaires, providing details on demographics, headache, AIWS characteristics and the occurrence of transient visual phenomena such as fragmented vision.

**Results:**

Of 808 migraine patients, 133 individuals (16.5%, mean age 44.4 ± 13.3 years, 87% women) reported AIWS symptoms throughout their lives. Micro- and/or telopsia (72.9%) were most frequent, followed by micro- and/or macrosomatognosia (49.6%), and macro- and/or pelopsia (38.3%), lasting on average half an hour. AIWS symptoms occurred in association with headache in 65.1% of individuals, and 53.7% had their first AIWS episode at the age of 18 years or earlier. Migraine patients with aura were more likely to report AIWS symptoms than those without aura (19.5% vs. 14.1%, *p* = 0.04). Participants with AIWS reported a higher incidence of 17 out of the 22 investigated visual phenomena.

**Conclusion:**

AIWS symptoms appear to be a common lifetime phenomenon in migraine patients. The correlation and clinical parallels between AIWS and migraine aura could indicate shared underlying pathomechanisms.

## Introduction

Alice in Wonderland Syndrome (AIWS) is considered [1] a rare clinical condition, primarily observed in children [2]. It is marked by a transiently altered visual and/or somatosensory perception of the body or surroundings [2–5], usually lasting 5 to 30 min [6, 7]. Additional symptoms may include alterations in perception of time, derealization, depersonalization, and somatopsychic duality (sense of being divided into two) [2–4]. Individuals experiencing AIWS remain aware of the illusory nature of the perception. The term was first introduced by John Todd in 1955[5] in reference to the homonymous novel by Lewis Carroll (Charles Lutwidge Dogson) and its main character Alice. He described perceptual disturbances in six patients, four of whom had migraine. Noteworthy, comparable alterations had been documented two years prior by Cro W. Lippmann [8], also in individuals with migraine. Since then, over 150 AIWS cases have been published in the literature with over 60 associated symptoms described in total. These include particularly diverse visual phenomena [2] like alterations in the perception of objects’ size (dysmetropsia) or shape (metamorphopsia).

In childhood, AIWS is associated most frequently with encephalitis caused by the Epstein–Barr virus and migraine [9, 10]. In adulthood, migraine is commonly suspected as the primary cause [2, 10, 11]. Other potential underlying conditions of the AIWS include various viral infections, epilepsy, intoxications and fever [10–12]. While AIWS can persist for years, complete remission is common. With chronic diseases like migraine, symptoms may recur during active phases (ictal) [2].

Tough standardized diagnostic criteria are lacking, efforts have been made to classify AIWS in more detail. In 2013, Lanska and Lanska [6] categorized 81 AIWS-cases based on the reported symptoms into three types: somesthetic- (Type A), visual- (Type B) and somesthetic and visual alterations in perception (Type C). Somesthetic alteration in perception involve distortions in perceiving one’s body or environment, like micro- and macrosomatognosia. Visual perception alterations include symptoms such as micropsia, macropsia, pelopsia, and telopsia. Type B was most prevalent (75%), primarily affecting children who had viral infections. Type C accounted for 16% of the cases, affecting mostly older patients with migraine. Building on this classification, in 2015, Mastria [10] designated Type A, B and C features as obligatory symptoms, while symptoms of derealization, depersonalization, somatopsychotic duality, and change in time perception were defined as facultative.

The underlying mechanisms of AIWS remain poorly understood. Its frequent connection to migraine, however, has sparked discussions about shared pathophysiological processes. Among individuals with migraine, visual aura is the predominant type of migraine aura [13]. Other migraine-associated phenomena like AIWS or visual snow also represent disorders of the visual system in which the occipital cortex is thought to be involved [10, 14, 15]. A potential association between migraine and AIWS might be explained by heightened cortical excitability and lack of habituation in individuals with migraine, increasing their susceptibility to AIWS. Other authors even propose that AIWS may be a manifestation of migraine aura [16]. In fact, the pathophysiological correlate of migraine aura, cortical spreading depolarization (CSD) and the thereby induced depression of spontaneous brain activity [17], may also play a role in the development of AIWS [16]. However, various hypotheses have been proposed, including alterations in functional connectivity in both migraine and AIWS patients [18].

Overall, epidemiology, clinical characteristics and pathophysiology of AIWS in migraine remain understudied. Here, we aimed to obtain insights into the prevalence and features of AIWS in migraine analyzing a large cohort of patients diagnosed with migraine with and without aura according to the International Classification of Headache Disorders 3 (ICHD-3) criteria [19]. We hypothesized AIWS symptoms to be more frequent in patients with migraine with aura compared to migraine without aura.

## Methods

### Study design, setting and participants

This work is part of a cross-sectional study among migraine patients with and without aura conducted at the Headache Center, Charité – Universitätsmedizin Berlin. Data were collected between August 2020 and March 2023 using a standardized questionnaire. For detailed methods please refer to the primary publication [20]. In summary, adult patients with migraine according to the ICHD-3 criteria were approached during their regular visit at the outpatient clinic and asked for consent to participate in the subsequent study. Patients with insufficient German proficiency, other known headache disorders than tension-type headache, regular use of five or more medications (polypharmacy) and/or severe psychiatric disorders that could influence the analyses were excluded. The questionnaire was filled out on an iPad on site.

### Study instruments

The questionnaire designed by the authors comprised four subunits to collect data on (i) demographics, (ii) headache characteristics, (iii) AIWS features, and (iv) visual phenomena. Data entry into an electronic database was facilitated through the use of REDCap software (REDCap 12.0.33-© 2022 Vanderbilt University, Nashville, TN, USA).

#### Demographic (i) and headache characteristics (ii)

Staff members recorded demographics (age, sex), ICHD-3 headache diagnosis, monthly headache and migraine days of the month prior to the questionnaire as well as headache specific medication. Patients subsequently provided additional information on headache characteristics (years lived with migraine, family history, migraine aura features) and completed the subunits (iii) and (iv) of the questionnaire.

#### Alice in Wonderland Syndrome (iii) and visual phenomena (iv)

The subunit iii) of the questionnaire started with a question to assess whether participants had experienced at least one core symptom of the AIWS [10] in their lifetime including: (1.) micropsia and/or telopsia (objects appearing smaller or farther away than in reality), (2.) macropsia and/or pelopsia (objects appearing bigger or closer than in reality) and/or (3.) micro- and/or macrosomatognosia (the body or parts of the body appearing smaller or larger than actual size). If participants indicated to have never had experienced any of the above summarized core symptoms, they proceeded directly to the next questionnaire (subunit iv – visual phenomena). If participants answered yes to at least one core symptom, further questions followed characterizing the AIWS in more detail. Those questions included time at first onset and last occurrence of AIWS symptoms along with accompanying symptoms, such as feeling of unfamiliarity/disconnection toward their own body/the environment, altered time perception, somatopsychic dualty, feeling of floating and (un)pleasant feeling during the symptoms. Furthermore, aspects like the awareness of the unreal nature of the perception, duration of the experience, and suspected underlying causes were collected. Patients were further asked whether they had ever experienced headache before, during or after the AIWS symptoms.

In the last part of the questionnaire, (part iv) participants were asked whether they had experienced different specific visual phenomena at some point in their life and whether these were associated with headache. Specifically, participants were required to differentiate whether they always, never or sometimes experienced visual phenomena in the context of headache. Alternatively, “I don’t know/ I am unsure” could be selected. The visual phenomena included deformed vision, hallucination, fragmented vision, bright light, blurred vision, zig-zag lines, one single scotoma, multiple scotomata, small bright dots, white dots, lines, geometrical shapes, water oil, half moon, hemianopsia, tunnel vision, oscillopsia, fragmented objects, corona, anopia, and negative film.

### Objective and endpoints

The primary objective of the study was to assess the prevalence of AIWS symptoms in migraine patients with aura as compared to those without aura. Additionally, a comprehensive descriptive analysis was carried out to gain insights into various aspects of AIWS characteristics. This analysis covered the nature of symptoms, duration, age at onset, emotions experienced during symptoms (joy/fear), awareness of the unreality of perception, the correlation of headaches with AIWS symptoms, accompanying symptoms, and the reported perceived causes by affected individuals.

Secondary objectives involved examining differences between the two groups of participants with and without AIWS. These comparisons were made in terms of the number of years lived with migraine, the categorization of migraines as episodic or chronic, and the presence of a positive family history for migraine. An additional exploratory endpoint focused on evaluating the prevalence of various visual phenomena in participants with AIWS compared to those without AIWS.

### Statistical analyses

For statistical analysis we used IBM SPSS Statistics (IBM SPSS Statistics ©; 23.0, for Mac). To minimize missing values, questions were predominantly designed to be mandatory before proceeding to the next section. Missing values are indicated for each analysis or can be derived from the adjusted overall sample size. We conducted descriptive analyses for demographic and headache characteristics as well as questions on AIWS features and visual phenomena. Categorical variables are reported as absolute numbers (n) and percentages (%), whereas numerical variables are displayed as mean values ± standard deviation. AIWS was considered present if patients affirmed at least one core symptom according to the proposed diagnostic criteria by Mastria [10]. For subsequent analyses, the AIWS cohort was then classified into Types A, B, and C following the criteria set by Lanska and Lanska [6]. To analyze differences in the frequency of AIWS-core symptoms between migraine with and without aura as well as different visual phenomena between migraine patients with and without AIWS-core symptoms, we performed chi-square tests. Multiple testing was corrected by applying the Bonferroni method. To assess whether migraine with aura could act as a confounder or effect modifier, a logistic regression model was employed. In the case of a confounder, we reported an adjusted measure of the association that accounted for the confounder, whereas for an effect modifier, the stratum-specific measure of the association was reported. For multiple-choice questions, the percentages indicated represent the relative proportion of the corresponding responses. Accordingly, the total value may exceed 100%.

## Results

### Prevalence of AIWS in migraine patients

The study population consisted of 808 migraine patients (87.0% female, 73.6% episodic migraine, 43.7% migraine with aura), with an average age of 44.4 years (± 13.3 years). A total of 133 participants (16.5%, mean age 41.5 ± 12.4 years, 89.5% women, 51.9% migraine with aura) reported to have experienced at least one AIWS-core symptom at some point in their life (AIWS cohort). Information about AIWS symptoms was not previously collected in the patients’ electronic records but was newly obtained through the questionnaire used in this study. Demographics of the overall migraine population and the AIWS cohort are summarized in Table 1.

**Table 1:** Study population and headache characteristics

	*n* (%) or mean ± SD			Missings *n* (%)	
	Total, 808 (100)	AIWS, 133 (16.5)	No AIWS, 675 (83.5)	Total	AIWS
Female sex	703 (87.0)	119 (89.5)	584 (86.5)	–	–
Age	44.4 ± 13.3	41.5 ± 12.4*	45.0 ± 13.4*	–	–
Episodic migraine	595 (73.6)	90 (67.7)	505 (74.8)	–	–
Migraine with aura	353 (43.7)	69 (51.9)*	284 (42.1)*	–	–
Visual aura	313 (88.7 of aura patients)	65 (94.2 of AIWS aura patients)*	248 (87.3 of no AIWS aura patients)*	–	–
Sensory aura	150 (42.5 of aura patients)	39 (56.5 of AIWS aura patients)*	111 (39.1 of no AIWS aura patients)*	–	–
Years with headache	23.7 ± 13.8	23.0 ± 12.6	23.8 ± 14.1	34 (4.2)	4 (3)
Years since diagnosis	15.2 ± 12.9	15 ± 12.2	15 ± 13.0	44 (5.4)	5 (3.8)
Positive family history	564 ± 69.9	98 (74.2)	466 (69.0)	1 (0.1)	1 (0.8)

*Statistically significant difference between participants with and without AIWS

Migraine patients with aura were more likely to report AIWS symptoms compared to migraine patients without aura (19.5%, 69/353 vs. 14.1%, 64/455; χ^2^(1) = 4.3, *p* = 0.04, φ = 0.07). However, a visual aura was not a pre-requisite to patients experiencing AIWS, with 48% of patients with AIWS not having migraine aura (*n* = 64/133). No differences were observed between the two groups of participants with and without AIWS with regard to the number of years lived with migraine, episodic/chronic migraine, or positive family history.

### AIWS characteristics

**Fig. 1:**
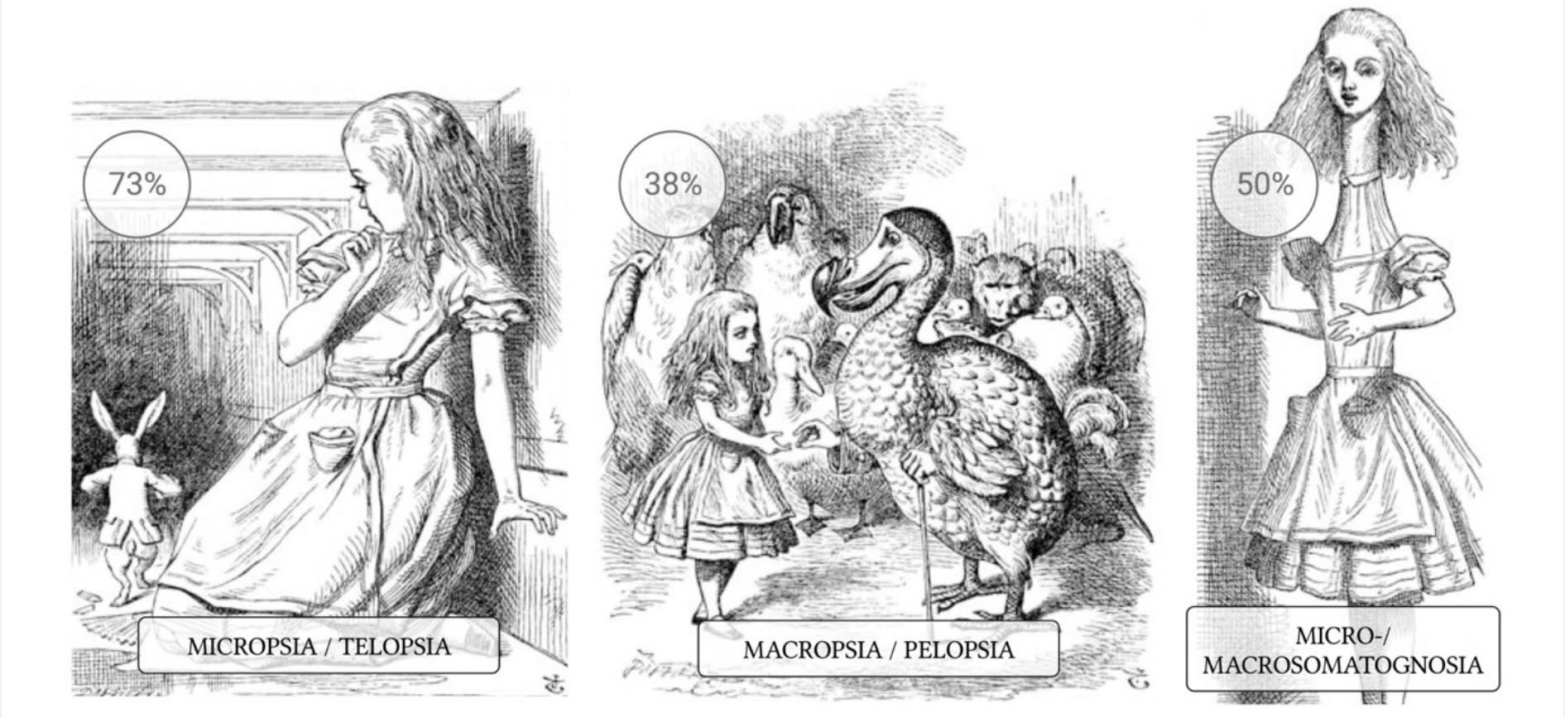
Prevalence of AIWS-core symptoms in patients with migraine. 73% micro- and/or telopsia; 38% macro- and/or pelopsia; 50% micro and/or macrosomatognosia. Created with *biorender.com.* Image in the public domaine, reprinted with permission [21]

Key**-**AIWS characteristics of study participants are summarized in Table 2. The average duration of symptoms was half an hour. Slightly more than half of the cases (52.9%, *n* = 64/121 (9 missing cases)) indicated the onset of AIWS symptoms at the age of 18 years or younger. Among the core symptoms outlined by Mastria et al. [10], micropsia and/or telopsia emerged as the most prevalent in our cohort (72.9%, *n* = 97/133) (Fig. 1). When categorizing migraine participants with AIWS into the three types proposed by Lanska and Lanska [6], Type B (involving visual illusions alone) was observed as the most prevalent (50.4%, *n* = 67/133). Type A (involving somesthetic perceptual symptoms) and Type C (involving both visual and somesthetic perceptual symptoms) were evenly distributed at around 25% each (Type A – 24.1%, *n* = 32/133; Type C – 25.6%, *n* = 34/133). Among participants with AIWS, 45.1% (*n* = 60/133) reported to have experienced more than one core symptom in their lifetime. Specifically, 29.3% (*n* = 39/133) reported encountering two out of the three core symptoms, while 15.8% (*n* = 21/133) reported to have experienced all three.

**Table 2:** AIWS characteristics

	*n* (%*) or mean ± SD	Missing *n* (%*)
*Cardinal symptoms*		
Micropsia and/or telopsia	97 (72.9)	
Macropsia and/or pelopsia	51 (83.3)	
Micro- and/or Macrosomatognosia	66 (49.6)	
Age at onset [years]	22.1 ± 14.08	12 (9)
Age at last event [years]	38.2 ± 13.2	7 (5.3)
Sense of joy/happiness at that moment	9 (6.8)	–
Sense of fear/dread at that moment	55 (41.4)	–
Awareness that perception is not real	108 (81.2)	–
Headache before/after/during	84 (65.1)	4 (3.0)
*Associated symptoms*		
Feeling of unfamiliarity/disconnection toward their own body, sensations, or physical experiences, perceiving them as unreal and distant	64 (49.2)	3 (2.3)
Environment appeared unreal, surreal, or inexplicably changed	70 (53.8)	3 (2.3)
Sense of time passing either faster or slower	81 (62.3)	3 (2.3)
Sensation of being two separate individuals simultaneously	21 (15.9)	1 (0.8)
Feeling of floating	51 (39.2)	3 (2.3)
Cause attributed by the participant for the perceptual changes	26 (19.7)	1 (0.8)
Sleep deprivation	13/26 (35.1^#^)	–
Migraine	5/26 (13.5^#^)	–
Traumatic experience, PTSD, dissociation, panic attack	4/26 (10.8^#^)	–
Emotions, stress	3/26 (8.1^#^)	–
Fever	4/26 (10.8^#^)	–
Drugs	2/26 (5.4^#^)	–
Others	6/26 (16.2^#^)	–

*of AIWS cases, ^#^% of answers

The three most frequently reported accompanying symptoms were alterations in the perception of time (62.3%, *n* = 81/130), feeling of unfamiliarity or disconnection from the environment (53.8%, *n* = 70/130), and a similar feeling toward their own body (49.2%, *n* = 64/130). The vast majority (81.2%, *n* = 108/133) were aware that the sensations were not genuine. AIWS symptoms were predominantly perceived as unpleasant, with 41.4% (*n* = 55/133) expressing anxiety and fear, while 6.8% (*n* = 9/133) reported feelings of joy or happiness. Almost two thirds (65.9%, *n* = 85/129) reported developing headaches either prior, during or after AIWS symptoms. Among participants with AIWS, 19.7% (*n* = 26/132) claimed to know the cause for the perceived disturbance in perception, with sleep deprivation (35.1%, *n* = 13/26 answers), migraine (13.5%, *n* = 5/26 answers), emotion/stress (8.1%, *n* = 3/26 answers) and fever (10.8%, *n* = 4/26 answers) being mentioned as the most frequent causes.

### AIWS and visual phenomena

Participants who experienced AIWS reported more often to have experienced 17 out of the 22 investigated visual phenomena compared to individuals without AIWS (see Table 3). Considering the observed increased prevalence of AIWS in individuals with migraine aura compared to those without aura, there is a possibility that the association between the 17 visual phenomena and AIWS could be influenced by the presence of migraine aura, either as a confounder (a variable causing distortion if unevenly distributed between groups) or effect modifier (a factor separating the sample into subgroups with different disease associations). In the majority of visual phenomena showing a correlation with AIWS (10 out of 17), the association with AIWS remained independent of a concurrent migraine aura diagnosis. For two visual phenomena (scotomata, geometrical shapes) showing a correlation with AIWS, migraine aura was identified as a confounding factor. Upon adjusting for aura as a confounding factor, the initial significant association was no longer present (see Table 3). In another four of the 17 visual phenomena with a correlation to AIWS (hallucination, fragmented vision, scotoma, white dots), migraine aura emerged as an effect modifier, requiring a separate analysis for migraine subgroups with and without aura. Following this subgroup analysis, the initially observed significant association between these four visual phenomena and AIWS persisted only for the subgroup of AIWS patients without aura, while no correlation was observed for the subgroup of AIWS patients with aura.

**Table 3:** AIWS and visual phenomena

AIWS vs. no AIWS		*p* value*	Confounder	*p* value**	Missings°
Deformed vision 46 (37.4%) vs. 71 (11.3%)	No deformed vision 77 (62.6) vs. 558 (88.7%)	< 0.001	–	–	10 (7.5%) vs. 46 (6.8%)
Hallucination 35 (28.0%) vs. 45 (7.2%)	No Hallucination 90 (72.0) vs. 581 (92.8%)	< 0.001	‡	No aura: < 0.001 Aura: 0.22	8 (6.0%) vs. 49 (7.3%)
Fragmented vision 17 (13.7%) vs. 30 (4.8%)	No fragmented vision 107 (86.3) vs. 596 (95.2%)	< 0.01	‡	No aura: < 0.001 Aura: > 0.999	9 (6.8%) vs. 49 (7.3%)
Bright light 82 (64.6%) vs. 271 (43.9%)	No bright light 45 (35.4) vs. 345 (56.0%)	< 0.001	–	–	6 (4.5%) vs. 59 (8.7%)
Blurred vision 100 (79.4%) vs. 377 (59.0%)	No blurred vision 26 (20.6) vs. 262 (41.0%)	< 0.001	–	–	6 (4.5%) vs. 59 (8.7%)
Scotoma 51 (40.8%) vs. 138 (21.6%)	No scotoma 74 (59.2) vs. 500 (78.4%)	< 0.001	‡	No aura: < 0.001 Aura: > 0.999	8 (6.0%) vs. 37 (5.5%)
Scotomata 27 (21.8%) vs. 69 (11.1%)	No scotomata 97 (78.2%) vs. 551 (88.9%)	0.03	†	0.44	9 (6.8%) vs. 55 (8.1%)
Small bright dots 85 (65.9%) vs. 288 (46.1%)	No small bright dots 44 (34.1) vs. 337 (53.9%)	< 0,001	–	–	4 (3.0%) vs. 50 (7.4%)
White dots 34 (27.6%) vs. 88 (14.3%)	No white dots 89 (72.4) vs. 526 (58.7%)	< 0,01	‡	No aura: < 0.001 Aura: > 0.999	10 (7.5%) vs. 61 (9.0%)
Geometrical shapes 25 (20.2%) vs. 62 (9.9%)	No geometrical shapes 99 (79.8) vs. 565 (90.1%)	0.02	†	0.22	9 (6.8%) vs. 48 (7.1%)
Water oil 58 (47.5%) vs. 143 (23.0%)	No water oil 64 (52.5) vs. 480 (77.0%)	< 0.001	–	–	11 (8.3%) vs. 52 (7.7%)
Hemianopsia 35 (28.7%) vs. 94 (14.9%)	No hemianopsia 87 (71.3) vs. 535 (85.1%)	< 0.01	–	–	11 (8.3%) vs. 46 (6.8%)
Tunnel vision 49 (40.5%) vs. 81 (12.9%)	No tunnel vision 72 (59.5) vs. 548 (87.1%)	< 0.001	–	–	12 (9.0%) vs. 46 (6.8%)
Oscillopsia 62 (50.4%) vs. 160 (25.3%)	No oscillopsia 61 (49.6) vs. 472 (74.7%)	< 0.001	–	–	10 (7.5%) vs. 43 (6.4%)
Fragmented objects 20 (16.8%) vs. 40 (6.5%)	No fragmented objects 99 (83.2) vs. 574 (93.5%)	< 0.01	†	0.04	14 (10.5%) vs. 61 (9.0%)
Corona 39 (31.7%) vs. 63 (10.0%)	No corona 84 (68.3) vs. 564 (90.5%)	< 0.001	–	–	10 (7.5%) vs. 48 (7.1%)
Negative film 13 (10.5%) vs. 22 (3.5%)	No negative film 111 (89.5) vs. 604 (96.5%)	0.02	–	–	9 (6.8%) vs. 49 (7.3%)

*p values corrected for multiple testing using the Bonferroni method; ** Adjusted *p*-values, stratified for confounding or effect modification, and corrected for multiple testing through the application of the Bonferroni method; ° "missing values" include participants who responded with "don’t know" or did not answer the question; † Aura identified as confounder in the logistic regression model; ‡ Aura identified as effect modifier in the logistic regression model

## Discussion

In this prospective, cross-sectional cohort study involving 808 migraine patients from a tertiary headache center, the lifetime prevalence of at least one AIWS-core symptom, considered as AIWS according to the proposed diagnostic criteria by Mastria [10], was 16.5%. Individuals with migraine with aura were more likely to have encountered AIWS symptoms compared to individuals without aura. Symptoms were predominantly experienced as unpleasant.

Epidemiologic studies on AIWS are rare and the few existing reveal inconsistent results [4, 10]. While one cross-sectional study with 3,224 high-school students indicated a lifetime prevalence of micropsia and macropsia of 6.5% in boys and 7.3% in girls [22], another study with 297 participants indicated a lifetime prevalence of 14% for micropsia, and 15% for macropsia [23]. Both studies did not record pre-existing or concomitant medical conditions, so that the proportion of migraine patients in these populations cannot be specified. Migraine, however, ranks as the second most common cause of AIWS in childhood and the most common in adulthood [5, 6, 10]. In line with this, our analysis revealed a high lifetime prevalence of AIWS symptoms at 17%. This corresponds to a recent prospective study on 210 migraine patients conducted at a specialized headache clinic indicating a lifetime prevalence of AIWS symptoms of 19% [24]. A different study on individuals with vestibular migraine revealed a prevalence rate of 14% [25]. Within the group of migraine patients, the frequency of AIWS symptoms appears to vary based on the presence of aura. In this study, migraine patients with aura were more prone to experience AIWS symptoms compared to those without aura. In a comparable study [24], among patients with AIWS symptoms, the rate of patients with aura was even higher (95%), whereas in our cohort a relevant proportion (48%) had no migraine aura.

Beyond the mere comorbidity, AIWS and migraine, particularly with aura, share several clinical similarities. First, onset of migraine attacks and AIWS symptoms appear to correlate in time, with 65% of AIWS patients reporting headache before, after or during AIWS symptoms. However, data on the frequency of the accompanying headache was not collected. Similarly, a study involving individuals with vestibular migraine revealed that AIWS distortions occurred in 77% of cases during the migraine episode and persisted throughout the attacks [25]. Second, AIWS and migraine aura exhibit similar temporal patterns: The average duration of AIWS symptoms in our study was reported to be around 38 min, closely mirroring the findings of Mastria et al. at 40 min [24] and other previous literature [6, 24]. This duration also roughly corresponds to the typical duration of a migraine aura, as indicated by earlier research [19] and our study population, where the most common duration for migraine aura was between 30 and 60 min (38.1%). We did not assess the temporal progression of AIWS symptoms. A gradual onset of symptoms would further strengthen the parallels between migraine aura and AIWS. Finally, both AIWS and migraine aura share visual and sensory disturbances as key symptoms. Consistent with previous studies [6, 24], visual perceptual disturbances were the most commonly reported AIWS-core symptom in our cohort, present in 50% of cases. However, also close to 50% of individuals also reported either solely somatosensory sensations (24%) or a combination of visual and somatosensory phenomena (26%). Also for migraine aura, visual disturbances predominate, but somatosensory and more complex symptoms may occur [13].

The intriguing parallels between AIWS and migraine aura have led to discussions about shared pathophysiological principles, with some authors proposing AIWS as a distinctive form of migraine aura [16]. One hypothesis suggests alterations in functional connectivity, especially in the visual cortex, underlying both conditions [18]. Changes in the functional connectivity especially within visual networks are well documented for migraine patients with aura [26, 27]. While the pathophysiology of AIWS remains poorly understood, lesion-based and functional MRI (fMRI) studies indicate the involvement of various cerebral regions [10] also including visual networks like the extrastriate visual cortex [14] as well as the temporal-parietal-occipital Carrefour [28], a meeting point of temporooccipital, parietooccipital and temporoparietal junctions, relevant for integrating visual and somatosensory information. A recent resting-state fMRI study [18] identified similarly reduced functional connectivity in the lateral and medial visual networks in AIWS patients and migraine patients with aura compared to healthy controls. These changes also affected areas like the lingual gyrus and the superior lateral occipital cortices known as "aura generator" [29], suggesting shared underlying pathophysiological processes. Individuals with AIWS, however, showed more extensive and noticeable changes in functional connectivity compared to those with migraine aura. In migraine patients, the extent of change in functional connectivity is associated with the complexity of migraine aura [30]. Therefore, the more pronounced changes in AIWS patients might be attributed to the more complex symptomatology of AIWS.

The complex perceptual disturbances in AIWS has led to the hypothesis of an impaired integration of multisensory stimuli [16]. In line with this hypothesis, Mastria et al. [16] detected increased inter-regional functional connectivity between the thalamus and four cortical areas in individuals with AIWS and typical migraine aura, compared to individuals with migraine aura alone, and healthy controls. Beyond shared alterations in thalamic connectivity, distinctive cortico-cortical changes specific to AIWS patients were observed. AIWS patients showed interictally an increased functional connectivity between the lateral occipital cortex (V3) and the posterior superior temporal sulcus, which is an important component of multisensory processing [31]. Interestingly, V3 is also implicated in the context of CSD [32]. CSD is characterized by a gradually propagating wave of near-complete breakdown of the transmembrane ion gradients and sustained neuronal and glial depolarization [33]. It spreads at a rate between 2-9 mm/min throughout the gray matter, independent of functional or vascular territories [17, 34]. Clinical evidence that the typical sequential symptoms of migraine aura are caused by CSD is based on measurements of regional cerebral blood flow or its surrogates [32, 35] and in one patient also on direct electrocorticographic recordings [36]. In the context of AIWS, it is interesting that CSD can trigger excitation and even electrographic seizures in surrounding cortex tissue into which it does not penetrate, both in humans and in animal experiments [37, 38], which could possibly trigger AIWS under certain conditions.

Despite these similarities, there are also findings that could suggest AIWS to be more than a complex migraine aura. In our study, migraine patients with AIWS reported significantly more often to have experienced 17 of 22 transient visual phenomena compared to migraine patients without AIWS. Notably, the association between AIWS and 10 of those 17 visual phenomena was unaffected by the diagnosis of a migraine aura. For four visual phenomena aura was identified to serve as an effect modifier. Subsequently conducted subgroup analyses separately for migraine with and without aura revealed that the statistical significance of the association between AIWS and those four visual phenomena persisted solely in patients without aura. This observation challenges the hypothesis that AIWS and migraine aura reflect the same underlying conditions and rather indicate that AIWS may represent a distinct phenomenon within the spectrum of migraine-related visual perceptual disturbances.

AIWS is often portrayed as a syndrome primarily occurring in childhood [6], with speculation that the developing child’s brain may be more susceptible to such perceptual distortions [39]. A systematic review characterizing a total of 170 cases reported that 78% of the affected individuals were under 18 years old [2]. The average age of the entire cohort was 15.5 years. In our study, only slightly more than half of the cases (52.9%) indicated that the onset of AIWS symptoms occurred at the age of 18 years or younger. Migraine patients are known to exhibit heightened sensitivity especially to visual input [40] and show lack of habituation [41, 42]. The lack of habituation is thought to be a symptom of an abnormal thalamo-cortical function [42], which has been shown to be altered in both individuals with migraine with aura and AIWS [16]. Consequently, the higher average age observed in our cohort of migraine patients compared to age recordings from epidemiologic studies without specific focus on migraine might be attributed to the fact that in the general population susceptibility to perceptual distortions decreases from childhood to adulthood, whereas individuals with migraine maintain high susceptibility even into adulthood.

To our knowledge, this is the first study to describe the patients’ feelings associated with the occurrence of AIWS symptoms in a larger cohort, which were unpleasant in 41% of cases. While isolated case reports have described AIWS symptoms as a source of anxiety or panic [43–45], in other instances the symptoms were perceived as non-threatening [46]. Our result indicates that the burden associated with AIWS symptoms might be substantial and underlines the necessity to further study its role in migraine.

Limitations of this study include potential recall bias and suggestive answers due to the survey methodology, the absence of follow-up data, and the potential bias associated with collecting data at a tertiary headache institute. This might have led to an overestimation of the real numbers in the general migraine population affecting the generalizability of our results. Furthermore, aside from migraine, AIWS symptoms have been associated with various diseases like epilepsy or can manifest independently of AIWS syndrome. Especially facultative symptoms of depersonalization and derealization are also often reported in functional neurological disorders (FNDs), which are a frequent comorbidity in migraine patients. Therefore, our results may potentially overstate the true prevalence, as patients might have encountered AIWS symptoms related to a medical condition other than migraine. At the same time, considering the common occurrence of derealization and depersonalization in FNDs, it appears important to inquire specifically about other AIWS symptoms in migraine patients with comorbid FNDs.

Another challenge poses the absence of universally applicable diagnostic criteria making comparisons across different studies difficult. Moreover, we did not record how often the AIWS symptoms occurred, how often they were accompanied by headaches and how they developed over time. Frequent occurrence especially in temporal association with headaches and slowly progressive symptoms would have been further clinical indications of a migraine aura.

## Conclusion

In this comprehensive prospective study, AIWS emerges as a frequent lifetime symptom in migraine patients, indicating that it may be underdiagnosed in this population. AIWS might serve as a mechanistic paradigm for the investigation of aura- and non-aura-associated migraine features. The observed association between AIWS and migraine with aura, coupled with the temporally linked onset of headaches and a similar time course, suggests the possibility of shared underlying mechanisms.

## Data Availability

The data that support the findings of this study are available from the corresponding author upon reasonable request.
